# Practice pathways, education, and regulation influencing nurse practitioners’ decision to provide primary care: a rapid scoping review

**DOI:** 10.1186/s12875-024-02350-3

**Published:** 2024-05-23

**Authors:** Norah Elvidge, Megan Hobbs, Amanda Fox, Jane Currie, Suzanne Williams, Karen Theobald, Melanie Rolfe, Claire Marshall, Jane L. Phillips

**Affiliations:** 1 Cancer and Palliative Care Outcomes Centre, School of Nursing, Queensland University of Technology, Brisbane, Australia; 2 School of Nursing, Faculty of Health, Queensland University of Technology, Brisbane, Australia; 3 Metro North Health, Redcliffe Hospital, Redcliffe, Australia; 4 Improving Palliative Care Through Clinical Trials (ImPaCCT), Faculty of Health, University of Technology Sydney, Sydney, Australia; 5 Children’s Health Queensland Hospital and Health Service, Brisbane, Australia

**Keywords:** Nurse practitioners, Scope of practice, Workforce, Health priorities, Primary healthcare

## Abstract

**Background/Objective:**

Initially established to improve access to healthcare, particularly for primary care, the full potential of the nurse practitioner role is yet to be realised in most countries. Despite this, most countries are working to meet an ageing population’s increasing healthcare needs and reduce healthcare costs and access disparities. Achieving these outcomes requires reform at multiple levels, including nurse practitioner practice pathways, education and regulation, and identifying the barriers and facilitators to optimising their primary care role.

**Methods:**

A rapid scoping review of nurse practitioner practice pathways, education and regulation inclusive of: (1) a systematic search of Medline and CINAHL for peer-reviewed English language articles, including opinion pieces published between January 2015 and February 2022; and (2) a web-based search of nurse practitioner program entry requirements of International Nurse Regulator Collaborative country members with a protected nurse practitioner title and prescribing rights, plus the Netherlands. The individually summarised search data was integrated and synthesised using Popay’s narrative approach.

**Results:**

Emerging evidence from the included nurse practitioner courses (*n* = 86) and articles (*n* = 79) suggests nurse practitioners working in primary care provide safe, effective care and improve healthcare efficiencies. However, different regulatory and educational models are required if the primary care nurse practitioner is to meet growing demand.

**Conclusions:**

International variations in entry criteria, curriculum, and regulation shape the global profile of the nurse practitioner primary care workforce and their practice setting. For countries to grow their primary care nurse practitioner workforce to meet unmet needs, different entry requirements, program content and accredited post-registration transitional programs must be urgently considered.

**Supplementary Information:**

The online version contains supplementary material available at 10.1186/s12875-024-02350-3.

## Background

Primary care is an essential foundation of effective and responsive healthcare systems as a person’s first point of access to healthcare and the source of referrals to other services [1]. Since the Alma-Ata declared primary care essential to all effective healthcare systems, primary care has been enshrined in numerous global policies [2]. However, effective primary care depends on an interdisciplinary partnership approach that integrates health services to meet people’s health needs. It also addresses the broader determinants of health through multisectoral policy and action and empowers individuals, families and communities to take charge of their health [3, 4]. 

While global healthcare has rapidly improved over the past three decades [5], and new technologies enable people to maintain their autonomy and function independently for longer in the community, there is a growing need for a well-prepared, diverse, and collaborative primary care workforce [6–8]. Global healthcare systems are increasingly required to manage a rapidly ageing population and a growing burden of complex illnesses (e.g., diabetes, chronic kidney disease and cardiovascular disease) [5] in an environment facing substantial health workforce shortages. The need for a skilled workforce to engage in primary and secondary prevention, screening, assessment, triaging and managing activities has never been greater [1, 9]. Without significant health workforce reforms, the ongoing improvement in the health and well-being of those living in high-income countries cannot be guaranteed [5]. 

With fewer physicians selecting primary care as a career, access to primary care is challenging, leaving many people with unmet healthcare needs. As a result, nurse practitioners (NPs) are increasingly being called upon to strengthen and improve healthcare access and performance, especially for underserved communities and those with complex care needs [1, 10, 11]. Several NP courses have responded to these changing epidemiological needs with the inclusion of specialist content to improve the management of common primary care concerns such as mental health conditions, diabetes mellitus and other common endocrine conditions [12, 13]. However, current regulatory and entry requirements for educational programs in some countries do not facilitate NPs undertaking primary care roles. For example, despite the NP role being explicitly established in Australia to increase primary healthcare access [14], very few of the 2,425 NPs [15] currently practice in primary care [16, 17]. Understanding the educational entry pathways and regulatory requirements and their impact on nurse practitioners’ provision of primary healthcare is critical to addressing this policy mismatch.

As the international healthcare system evolves, and demands increase, it is timely to examine the benefits and limitations of international models of regulation and education on the composition of the NP workforce, and to consider their applicability to shaping the NP workforce of the future.

## Aim

To examine the international nurse practitioner practice pathways, education and regulation that prepare nurse practitioners for primary care roles across high-income countries with protected nurse practitioner titles.

## Methods

### Design

A rapid scoping review including: (1) a web-based review of the international entry requirements of approved NP programs and (2) a review of the peer-reviewed literature. This review sought to determine the international practice pathways, education and regulation that prepare NPs for a primary care role. A rapid review design was adopted to generate an expedient synthesis of multiple sources of evidence, which facilitated streamlining the search strategy, data extraction and bias assessments [18–20]. 

### Review 1: Web-based search of the international requirements of NP programs

#### Inclusion criteria

The International Nurse Regulator Collaborative member countries with a protected ‘nurse practitioner’ title; or other high-income countries with country or jurisdiction-level nursing boards responsible for nurse practitioners’ endorsement, licensure or registration (‘endorsement’); and who require candidates to have completed an accredited Master’s or Doctor of Nursing Practice degree, with mandated supervised clinical placement hours and a dissertation.

Public website pages published in English detailing NP programs from eligible countries with less than 99 NP programs were searched and included. In the United States of America (US), with ≥ 100 NP programs, the 10 top NP programs from the ‘Best Nursing Schools Rankings’ [21] plus the top 20 rankings from central [22] or rural states [23] delivering NP programs were included. This sampling approach captured geographical, socioeconomic and cultural diversity in university rankings and NP program size.

Google searches were conducted between 1st March and April 2022 using i) country name, ii) ‘NP’ or equivalent, and iii) ‘program’, ‘university’, or equivalent local term. If the respective jurisdictional standards for practice were not included on the identified university website, additional country-level Google searches were conducted using “nurs* standards” and the respective country’s name.

#### Data extraction

Two reviewers (MR & CF) extracted data into a purpose-built proforma that captured: the host organisation, web page and URL, program specialisation, delivery mode (e.g., online or in-person), entry requirements (e.g., clinical hours and clinical specialisation) and supervised clinical hours. The average clinical experience hours required to be eligible to enter a NP course and the average clinical supervision hours for each NP course were calculated for each included country. If clarification or additional information was required, the relevant member country contacts were emailed.

### Review 2: Rapid systematic review of NP regulation, education and practice evidence

The rapid systematic review was designed to answer the following search question: What are the practice pathways, education and regulation that prepare NPs for a primary care role?

#### Inclusion criteria

Eligible publications were (i) peer-reviewed; (ii) published in English between 01/01/2015 and 23/02/2022; (iii) reported empirical quantitative or qualitative data or presented an expert opinion on NP practice pathways, education and regulation; and (iv) undertaken in the included countries as detailed in Part 1 of this review. Publications that (i) did not address the search questions; (ii) focused on outdated NP legislation; (iii) countries other than those identified in Review 1; (iv) reviewed literature published outside of the study time frame; or (v) did not focus on primary care; or (vi) were conference abstracts; or (vii) study protocols, were excluded.

### Information sources and search terms

On 23rd February 2022, CINAHL and Medline via EBSCOhost were searched using the pre-defined strings (Refer to Additional File 1). The reference lists of included publications were hand-searched for other relevant studies and grey literature.

### Study selection and data collection

After the identified citations were imported into Covidence [24], one reviewer (NE) assessed eligibility before the other reviewers (NE, MH, MR and CF) extracted the (i) author list, (ii) publication year, (iii) country and (iv) key study findings into an electronic proforma.

### Risk of bias assessment

While the quality of each study was not assessed, the level of evidence was determined as per the method for grading guideline development recommendations [25]. 

### Synthesis and integration

A narrative synthesis [26] was used to integrate the different data sources to answer the search questions. After the data was extracted it was tabulated, counted and mapped to the key concepts, which helped to highlight the key outcomes and linked the emerging evidence [26]. This process allowed for visual representation of the data, and its alignment to: NP practice pathways, education and regulation. This process was led by one author (NE) before being reviewed by the senior author (JP) before being confirmed by other members of the team [26]. 

### Ethical approval and registration

As this was a review of existing literature and publicly available information, it was exempt from human ethics review.

## Findings

### Review 1: web-based search of the entry and clinical practice requirements of NP courses internationally

Eighty-six approved NP courses from seven countries, including six of the eight International Nurse Regulator Collaborative member countries: Australia, Canada, Ireland, New Zealand, Singapore, the United States (US), and the Netherlands, were included (Refer Additional File 2). A high-level summary of these NP programs’ entry and program requirements is provided (Refer to Table 1).

### International pathways into NP primary care practice

In general, NP courses are available to Registered Nurses with a Bachelor of Nursing, with Ireland, preferring an honours, but this is not compulsory. The exception is Australia, where a postgraduate nursing qualification and approximately 4.22 years of full-time equivalent clinical experience are required for admission to a NP course.

**Table 1:** Country-level summary of the NP clinical experience and specialisation requirements for program entry and endorsement

NP Programs/Country (*N* = 86)	Entry requirements			Program requirements			
	Years of previous clinical experience		Specialisation^a^	Required clinical hours		Defined clinical streams	
	*Range*	*M (SD)*	%	*Range*	*M(SD)*	*n*	%
Australia (*n =* 9)	4–5	4.22 (0.44)	100%	300–500	327.78 (66.67)	0	0%
Canada (*n* = 21)	2–3	2.10 (0.30)	0%	730–794	751.83 (27.37)	3^c^	100%
Ireland (*n* = 8)	1.5-2	2	100%	500–596	519.20 (42.93)	0	0%
Netherlands (*n* = 10)	2	2.00 (0)	100%	N/A^b^	N/A	2^d^	100%
New Zealand (*n* = 7)	2–3	2.86 (0.38)	0%	300	300 (0)	0	0%
Singapore (*n* = 1)	5	5.00 (0)	0%	500 w/ 12mth placement		0	0%
US (*n* = 30)							
Postgraduate certificate (n = 24)	0–3	1.29 (0.38)		300-1,000	565.50 (122.32)		
Masters (n = 21)	0–2	1.04 (0.42)		518.5-1,280	675.51 (167.67)		
Doctor of Nursing Practice (n = 22)	0–2	0.90 (0.50)		540-1,180	982.71 (154.17)		
Overall US trend	0–3	1.10 (0.45)	24%	300-1,280	363.73 (487.89)	8^e^	100%

^a^ % of NP programs that require evidence of clinical specialisation as an entry requirement; ^b^ Clinical hours embedded in program w/ total number of hours not specified; ^c^ Paediatric/Adult/Family; ^d^ Mental health/Physical health; ^e^ Adult-Gerontology (Acute), Adult-Gerontology (Primary), Paediatrics (Acute), Paediatrics (Primary), Psychiatric/Mental Health, Women’s Health, Family Practice (Generalist in Primary Care), and Neonatal

### Review 2: rapid systematic review of NP regulation, education and practice pathways

**Fig. 1:**
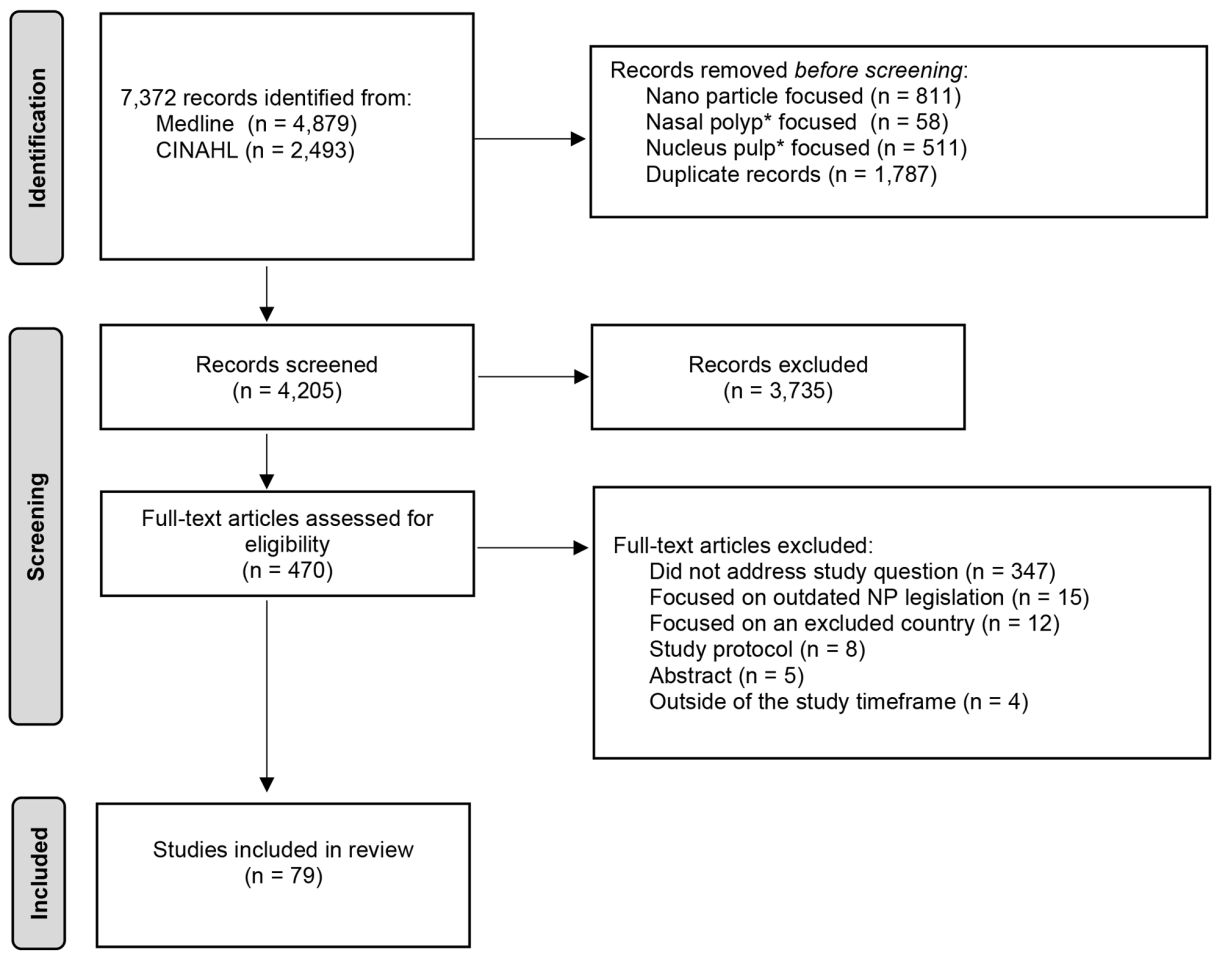
PRISMA flow chart of the rapid systematic review of the literature on NP regulation, education and practice evidence

The original search yielded 7,372 articles (Refer to Fig. 1), with 1,380 irrelevant articles (largely related to nanoparticles or nasal polyps) and 1,787 duplicates removed, leaving 4,205 publications titles and abstracts that were screened by a single reviewer (NE). Of the 470 publications that went for a full-text review, 79 were included in the final review. A brief summary of findings of the included articles can be found in Table 2.

**Table 2:** Summary table of included articles and relation to review foci

Reference Details			Foci		
Author/ Year/ Country	Design/ Level of Evidence	Summary	Pathways	Education	Regulation
Bin Abdul Baten and Wehby (2022), USA	Secondary analysis of service data (Level IV)	An analysis of the impact of the Affordable Care Act (ACA) Medicaid expansion on NP practice found that impoverished patients in states where NPs have full practice authority had greater access to primary healthcare compared to those living in states where NPs had a reduced scope of practice (*p* < 0.05).			**X**
Cuccovia, Heelan-Fancher (2022), USA	Expert Opinion (Level VII)	Extending NPs’ full practice authority in Massachusetts during COVID-19 demonstrates the feasibility and utility of permitting NPs to practice to their full potential, resulting in legislation changes allowing this to continue.			**X**
Smith (2022), USA	Secondary analysis of service data (Level IV)	A 6.5% increase in NPs with full scope of practice authority who provided visits and billed independently (*p* = 0.03). No evidence that relaxing the scope of NP practice laws changed the volume or allocation of patients to NPs. Autonomous practice may reduce healthcare costs as NPs receive a lower reimbursement for their primary care services.			**X**
Want, Goodman (2022), USA	Observational Survey (Level VI)	Primary care NPs (*N* = 102) who received education in a specific skill were more likely to perform it than those without education (*p*= 0.001–0.034), and suburban and rural NPs were more likely to perform specific skills than urban NPs (*p* = 0.005–0.039).		**X**	
Park, Faraz Covelli (2022), USA	Cross-Sectional Survey (Level VI)	10% of primary care NPs (*N* = 8400) completed postgraduate training or education. Those who completed residency training were more likely to treat underserved populations, reported increased confidence, team collaboration and role satisfaction, and were less likely to want to leave their roles.		**X**	
Germack, Harrison (2022), USA	Mixed Methods Study (Level VI)	Of the primary care NPs from 6 US states (*n* = 1244) with different scope of practice laws: 15% worked in rural communities, and these NPs were more likely to hold a Family NP certification. Rural NPs were more likely than urban NPs to manage their own patient panel independently (56% vs. 43%; *p* < 0.01).	**X**		
Lavoie and Clarke (2022), USA	Qualitative Study (Level VI)	NP educators’ (*N* = 27) perceptions of the new direct entry NP program pathways for novice RNs or non-nurses felt students reached similar levels of competence by program completion despite starting from different bases, leading the authors to conclude that continuing multiple entry routes into NP programs is advisable.	**X**		
Adams and Carryer (2021), New Zealand	Qualitative Study/Policy Analysis (Level VI)	Discussion on how despite a national requirement to expand primary care services; structural health sector changes, a competitive contractual environment, and policy and sector support of privately owned physician-led general practice is restricting the establishment of NP services.			**X**
Gardner, Helms (2021), Australia	Mixed Methods Study (Level VI)	Identifies NP core competency by meta-specialty as follows: Chronic and complex care, Ageing and Palliative Care, Emergency and Acute Care, Child and Family Health, Mental Health, & Primary Health.		**X**	
Mounayar and Cox (2021), USA	Expert Opinion (Level VII)	Evaluation of a residency programs for novice primary care NPs (*n* = not reported) found that they lacked confidence compared with novice primary physicians. Suggestion that residency programs may help to build confidence, reduce turnover in primary care NPs and improve patient outcomes, and recommends that there is universal accreditation and expansion of residency programs.		**X**	
Barnett, Balkissoon (2021), USA	Systematic Review (Level V)	Findings from the 11 included studies revealed primary care NPs provide quality care, equal to, or superior to that of physicians. Leading to a recommendation that state legislation be changed to enable full practice authority for NPs.			**X**
Timmons, Norris (2021), USA	Secondary analysis of service data (Level IV)	Expanding NPs’ scope of practice from ‘restricted’ to ‘reduced’ would increase 116,742 total care days delivered annually to Medicaid patients in New York (*p* = 0.84), and expanding the scope of practice to full practice authority would lead to a statistically significant increase of 842,284 total care days annually for Medicaid patients (*p* = 0.02). Recommendations were made to expand NPs’ scope of practice to full practice authority.			**X**
Neprash, Smith (2021), USA	Secondary analysis of service data (Level IV)	While there was no difference in the complexity of care, compared to urban primary care NPs, rural NPs have greater clinical autonomy, are more likely to be the patient’s sole provider of care, prescribe Schedule II controlled medications, and practice with less physician oversight. The clinical complexity of primary care patients managed by NPs did not differ between rural and urban settings.	**X**		
Gibson, Pravecek (2021), USA	Program Evaluation (Level VII)	A two-year preceptorship program to prepare Family (generalist) NP graduates for rural practice (*n* = not reported), composed of workshops and simulations to build rural skills, rural-based placements and financial incentives, was positively evaluated and led to 56% accepting a rural NP role (no comparison number given).		**X**	
Martsolf, Komadino (2021), USA	Cross-Sectional Survey (Level VI)	No significant differences in primary care NPs who were Doctor of Nursing Practice (DNP) prepared (*n* = 117) or Master-prepared (*n* = 1148) in their practice environments; however, the DNP-prepared NPs on average, spent more hours in leadership tasks and fewer in direct clinical care compared with the MSN-prepared NPs.	**X**		**X**
McNelis, Dreifuerst (2021), USA	Cross-Sectional Study (Level VI)	Family NPs (*N* = 3946) identified mental health assessments, ordering diagnostic tests, primary care procedures and managing chronic pain were missing from their clinical practice experience.		**X**	
Smith, Holland (2021), USA	Descriptive Pre-Post Study (incomplete) (Level VI)	Evaluation of a three-phase virtual simulation approach teaching and assessing basic primary care procedures delivered to rural NP students (*n* = 5) and rural NPs (*n* = 21). 15 participants (*n* = 4 NP students, *n* = 11 NPs) completed all three phases and reached the desired competency. While the post-test evaluation did not proceed as planned, participants expressed satisfaction with the process.		**X**	
Woroch and Bockwoldt (2021), USA	Observational Survey (Level VI)	Nearly half (47%, *n* = 94) of Family NPs (*N* = 198) < 5 years post-graduation learnt most common skills on clinical placement, 42% (*n* = 83) reported they learned their skills on the job post-graduation, and 61% (*n* = 121) did not feel adequately prepared to complete all procedures within their scope on graduation.		**X**	
Nikpour and Broome (2021), USA	Systematic Scoping Review (Level V)	Evaluating the impact of NPs’ scope of practice (SOP) on the management of chronic pain and opioid use disorder between 2008–2019 identified that: (i) state-based restricted/reduced SOP regulations were a primary barrier to managing chronic pain and opioid use disorder; and (ii) NPs feel prepared to manage chronic pain patients using opioids.			**X**
Currie, Carter (2020), Australia	Expert Opinion (Level VII)	Identifies the barriers to generalist NP development in Australia and recommends removing specialist prerequisites to study, standardised streams of education reflecting Australia’s national priorities (including primary health) and replacing hours of advanced practice prerequisites with competency assessments as strategies to increasing primary care NPs.	**X**	**X**	
Conrad, Burson (2020), Ireland	Literature and Policy Review (Level VII)	A collaboration with US stakeholders (*N* = not reported) suggested that the DNP could help expand Ireland’s NPs practice capacity. NPs currently work primarily in hospitals, and there is an opportunity and willingness for academic institutions to develop primary care partnerships to promote primary care practice among NPs in Ireland.	**X**	**X**	**X**
Clifford, Lutze (2020), Australia	Mixed Methods Study (Level VI)	NPs in urban (*n* = 16) and rural (*n* = 11) New South Wales, Australia, identify aged care, chronic/complex care, mental health, generalist/rural and remote, palliative care, diabetes and women’s health as primary care priority areas for NPs; and the need for generalist NPs education to position the NP workforce to meet these demands.	**X**		
Black, Fadaak (2020), Canada	Qualitative Observational Study (Level VI)	Canadian NPs and stakeholders (physicians, nursing and medical professional associations, patient representatives, and government officials) (*n* = 15, breakdown not disclosed) working in Alberta’s primary healthcare system reported on the ‘Nurse Practitioner Support Program’ policy and called for policy adjustments that enable NPs to access funding to improve financial viability, and for NPs to provide greater input into how their role can be better utilised.			**X**
Jeongyoung, Xinxin (2020), USA	Secondary analysis of service data (Level IV)	Extending NP scope to full practice authority, including prescriptive authority, significantly increased the number of primary care NP consultations (*p* < 0.05) required to improve primary care access.			**X**
Torrens, Campbell (2020)	Scoping Review (Level VII)	The leading barriers to primary care NP practice are poor awareness of their role and scope, interpersonal and reporting relationships, and restricted/reduced scopes of practice. Adaptability, collegiality, training, and education all facilitated the implementation of the NP role in primary practice.			**X**
Xue, Mullaney (2020), USA	Secondary analysis of service data (Level IV)	Between 2010–2016 the proportion of adults reporting a primary care NP as their usual source of healthcare increased, with the greatest increase in states where NPs had full practice authority compared to states with restricted practice.			**X**
Mileski, Pannu (2020)	Systematic Review (Level V)	Narrative evidence suggests that primary care NPs reduce unnecessary hospitalisations and improve healthcare access and patient outcomes among aged care facility residents, with the authors concluding that expanding the NP scope of practice to full authority is justified.	**X**		
Brommelsiek and Peterson (2020), USA	Mixed Methods Study (Level VI)	NP students (*N* = 16) who completed a 16-week immersive course inclusive of rural culture, health literacy, patient advocacy, interpersonal communication, patient management education, resilience and self-care training and rural placement reported it improved their capabilities and confidence.		**X**	
Bourque, Gunn (2020), Canada	Expert Opinion (Level VII)	British Columbia facilitated a stronger NP leadership presence in rural and remote communities by formally integrating NPs into the existing medical framework and establishing NP support programs and appraisal processes.	**X**		
Kaplan, Pollack (2020), USA	Observational Study (Level VI)	NP educators (*N* = 157) found that a crucial motivation for rural nurses entering a primary care NP program was ensuring flexible attendance with hybrid education and placements in the students’ communities, where possible. However, competition with other NP and non-NP programs for rural clinical placements is a major barrier.	**X**		
Jeffery, Donald (2020), Various	Comparative Analysis (Level VII)	A comparison of university programs (*N* = 24) identified an inverse relationship between the number of clinical hours required for admission and the number of clinical hours embedded in the program across the USA, Australia, Canada, Finland, Norway, and the Netherlands.		**X**	
Martsolf, Gigli (2020), USA	Expert Opinion (Level VII)	The “Consensus Model for APRN Regulation” is suggested to standardise the education and certification of specialty NP training. As NP specialisation in the USA rises, and < 30% of NPs consider their role as primary care, despite 70% of NP graduates being Family-certified NPs. A lack of standardisation of competencies and endorsement standards across non-accredited specialty programs contributes to this reality.	**X**		
Beeber, Palmer (2019), USA	Mixed Methods Study (Level VI)	DNP program directors (*N* = 130) report that 11% of DNP graduates work in primary care settings. DNP employers (*N* = 23) reported that DNPs were more likely to enter administrative or leadership roles than MSN-prepared NPs, who were more focused on clinical care than system change and policy.			**X**
Boris, Hoover (2019), USA	Expert Opinion (Level VII)	Contrasts core Veteran’s Affairs (VA) Primary Care Education competencies with the Quality and Safety Education for Nurses (QSEN) competencies to identify methods to improve leadership in the VA quality and safety curriculum. Local competency protocols need to improve in areas addressing informatics and safety to enhance leadership in quality and safety.		**X**	
Mundinger and Carter (2019), USA	Program Analysis (Level VII)	Of the accredited USA DNP programs (*N* = 553) offered between 2005–2018, 85% were non-clinical (leadership or administration), and 15% focused on advanced clinical practice, with the greatest growth in the non-clinical DNPs compared to clinical DNPs and MSN-prepared NP program (88% vs. 8%), which has affected NP practice context and has longer-term implications for the NP workforce.			**X**
Bellflower and Likes (2019), USA	Expert Opinion (Level VII)	Primary care needs are increasing in the US, yet 85% of DNP programs are non-clinical. Concerns that the DNP, without advanced clinical practice, is insufficient to prepare the NP workforce for safe and competent clinical care. While clinically focused DNP programs are more resource-intensive, they better meet patients’ healthcare needs.			**X**
Hudspeth and Klein (2019), USA	Policy Review (Level VII)	A clear role definition and an understanding of the scope of practice regulation are essential for safe practice. Variations in the US states’ scope of practice can unintentionally result in NPs breaching regulation scope, leading to a call for the states to work towards standardised regulation and full authority practice to provide NPs with additional employment opportunities across state lines and improve access to care.			**x**
Perloff, Clarke (2019), USA	Secondary analysis of service data (Level IV)	2012–2013 service usage data found in US states where NPs have full practice authority, there are higher rates of breast cancer screening (*p* < 0.0001) and fewer unnecessary ambulatory care-sensitive hospitalisations (*p* < 0.0001) than in reduced/restricted practice states. However, this should not be solely attributed to NP scope of practice, because this pattern of results also characterises the service use patterns of those cared for by primary care physicians.			**X**
Wolff-Baker and Ordona (2019), USA	Expert Opinion (Level VII)	Population ageing and rising chronic disease make NPs well-positioned to provide home-based primary care services. However, NPs’ inability to certify, recertify and sign orders for fee-for-service home health visits or certify terminal illnesses are barriers to care and increase healthcare costs, requiring reform of the legal definition of a ‘physician’.			**X**
Holland, Selleck (2019), USA	Program Description (Level VII)	Describes a concurrent educational program for NP candidates (*n* = 65 enrolled, *n* = 47 graduated) on a rural primary care specialty track without findings.		**X**	
Currie, Chiarella (2019), Australia	Realist Evaluation (Level VII)	The limited number and the non-investigative nature of the Medicare Benefits Schedule (MBS) items available to private practice NPs and the need for collaborative agreements are significant challenges to NPs’ scope of practice.			**X**
Cimiotti, Li (2019), USA	Observational Survey (Level VI)	NPs working in acute (*n* = 1,263) and primary care (*n* = 2343) across four US states describe professional and organisational factors associated with NP care quality. Scope of Practice was not associated with measures of clinical quality. Policies that create supportive environments offer the best potential to improve NP care and patient outcomes.			**X**
Kraus (2019), USA	Qualitative Study (Level VI)	Primary Care NPs (*N* = 15) identified concerns regarding nurses entering NP programs with insufficient clinical experience and basic nursing skills.	**X**	**X**	
Adams and Carryer (2019), New Zealand	Qualitative Study (Level VI)	NPs (*N* = 13) and NP candidates (*N* = 4) identified facilitators and barriers to becoming a rural primary care NP, which included a lack of nursing leadership, NP role perception, inconsistencies in training, practicums and costs. Facilitators included: commitment to NP development and mentorship, recognition of NP contribution to primary care and use of clinical assessment and decision-making skills.	**X**		
Cappiello, Simmonds (2019), USA	Cross-Sectional Survey (Level VI)	NP program directors (*N* = 24) capture the characteristics of their transition-to-practice programs. Programs existed in urban and non-urban settings; cared for a wide variety of patients; each service saw a median of 75,000 and 100,000 patients annually; most services offered rotations to specialist areas; and all provided onsite didactic education and training. Authors suggest that NP transition-to-practice programs are a growing and permanent trend.		**X**	
Painter, Sebach (2019), USA & Australia	Program Evaluation (Level VII)	Development, implementation and evaluation of a primary care NP (*N* = 15) residency program, with high satisfaction with all stakeholders, met its financial and workforce objectives. Uniform boarding has the potential to minimise role strain and improve job satisfaction.		**X**	
Faraz (2019), USA	Cross-Sectional Study (Level VI)	A USA national sample of NPs working in primary care (*N* = 117) found that 65% had completed a Family NP program. 23.2% worked in private practice, 18.1% in a federally qualified health centre, 9% in a hospital outpatient setting, and 7.9% in primary care in private practice. Focusing on the transition from education to practice is crucial to increase retention of primary care NPs, ultimately improving continuity of care and patient outcomes.	**X**	**X**	
Owens (2019)	Scoping Review (Level VI)	Qualitative evidence (*N* = 145 studies) found that rural primary care NPs who experienced a poor transition into their role are likelier to leave the profession within the first year. Six studies identify prior experience as an RN, NP orientation, and mentoring as key elements to a successful transition into a rural primary NP role.		**X**	
Schallmo, Godfrey (2019), USA	Systematic Review (Level V)	Evidence that NPs lack confidence and education preparation in selected advanced diagnostic and therapeutic skills. Programs are not teaching all the skills required of FNPs. Further exploration is required to quantify the most common skills and modify the curriculum accordingly.		**X**	
MacKay, Glynn (2018), USA	Mixed Methods Study (Level VI)	Very few (< 4%) of NPs (*N* = 159) reported participating in a residency program, with > 50% feeling unprepared for the first year of practice; 80% indicated they would have been interested in a residency program had it been available. Most reported that their university programs were rigorous but could not have prepared them for primary care. They suggested that “on-the-job” mentoring was required to navigate complex patient decision-making.		**X**	
Lowe, Jennings (2018), Australia	Cross-Sectional study (Level VI)	Care note data identified that NPs carry out diagnostic and pathology testing requests, referrals, and interventions relevant to their specialty in practice. NP scope of practice evolves according to their role and the clinical setting.		**X**	
Ritter, Bowles (2018), USA	Expert Opinion (Level VII)	Legally required NP supervision acts as a barrier to entry to practice and increases the cost of care for providers and patients.			**X**
Carter, Moore (2018), Global perspective	Expert Opinion (Level VII)	NPs are a suitable solution to a global shortage of generalist providers. A review of the USA’s 50 years of generalist NP practice identified laws and regulations that might impede NP practice and suggests greater availability of digital curricula for general practice NPs delivered through local universities.			**X**
Currie, Chiarella (2018), Australia	Cross-Sectional Survey (Level VI)	A survey of privately practising Australian NPs (*N* = 73) suggests that the 2010 NP policy changes have increased access to care, with most private NPs providing direct patient care (96%), including patient education and health promotion consultations (95%), the prescription of medications (95%), referrals to diagnostic investigations (92%); and 88% were responsible for diagnosing patients. However, only 59% reported working to their full scope of practice.			**X**
Xue, Kannan (2018), USA	Secondary analysis of service data (Level IV)	Per capita, primary care NP ratios increased between 2009–2013 in rural areas and counties with primary care workforce shortages. States, where NPs have full authority had a higher NP supply in rural and primary care settings. A better understanding of how legislative and regulatory decisions influence the supply of NPs may help inform health policy and address disparities.			**X**
Hicks, Rico (2018), USA	Qualitative Study (Level VI)	NP postgraduate program directors (*N* = 11 across nine postgraduate training sites) found novice NPs are seeking opportunities to complete residencies/fellowships in primary and specialty care. Evidence of the quality of these postgraduate programs is limited, yet suggests employer-sponsored postgraduate training programs support a new and competent generation of primary care NPs.		**X**	
Rugen, Dolansky (2018), USA	Program Evaluation (Level VI)	A self-and-mentor evaluation of NPs (*N* = 38) found a primary care residency program significantly improved clinical competency, leadership competency, interprofessional team collaboration, shared decision-making, and sustained relationships and quality improvement/population management throughout the residency year (*p* < 0.0001).		**X**	
Leidel, Hauck (2018), Australia	Qualitative Study (Level VI)	NP employers (*N* = 23) perceive the scope of NP practice as a workforce evolution rather than being tied to their endorsement with a specific patient population. Employers identified benefits and barriers to employing NPs. Employers sought NPs who were willing to fill the specific service gaps.			**X**
Rugen, Harada (2018), USA	Program Evaluation (Level VI)	NPs (*N* = 38) reported a year-long residency program in primary care improved diagnostic capabilities, planning care, leadership engaging others, improving supporting health systems and more.		**X**	
Nash, Hall (2018), USA	Comparative Study (Level VI)	An interprofessional education program between primary care NPs and dental students (cohort 1 *n* = 75, cohort 2 *n* = 116) improved self-efficacy in working within an interdisciplinary team compared with students who did not participate (*p* = 0.02).		**X**	
Traczynski and Udalova (2018), USA	Secondary analysis of service data (Level VI)	As NP autonomous practice increases primary care consultations, improves the quality of care, and decreases ED attendance, expanding NPs’ SOP would reduce administrative costs for physicians and NPs and indirect costs for patients.			**X**
Harper, McGuinness (2017), USA	Expert Opinion (Level VII)	NP residencies provide an opportunity for professional development for new NPs. However, the content of the available programs is varied, and they are not mandated. Qualitative graduate data suggests that residencies improve knowledge outcomes and team-based skills for primary care NPs.		**X**	
Cashin, Theophilos (2017), USA & Australia	Systematic Review (Level V)	There are legislative barriers in the USA and Australia to NPs functioning at their full scope of practice, including inconsistencies between states; lack of uniformity for licensure and titling (USA only); differing credentialing requirements (USA only); and requirement for collaboration or supervision with a medical doctor.			**X**
van der Biezen, Derckx (2017), The Netherlands	Qualitative Study (Level VI)	Primary care service managers (*n* = 7) and general practitioners (*n* = 32) reported they employ physician assistants/NPs to reduce GP caseloads, increase the practice’s capacity, improve quality of care, and deliver additional services. The authors suggest standardising NP and physician assistant roles and assisting policymakers to improve the skill mix in primary care.			**X**
Poghosyan, Liu (2017), USA	Observational Survey (Level VI)	This survey of primary care NPs (*N* = 314) identifies that increased organisational support for independent practice increased NP’s capacity to practice as primary care providers.			**X**
Brooks Carthon, Sammarco (2017), USA	Secondary analysis of commercial data (Level IV)	Linear regression models compared retail clinic opening rates between 2006 and 2013 in Pennsylvania, following scope of practice legislation changes with bordering states. An association was reported between relaxing practice regulations and retail clinic growth, as evidenced by a significant growth rate in net retail clinic openings per capita (*p* = 0.046) in Pennsylvania and non-significant growth in the two bordering comparator states.			**X**
Helms, Gardner (2017), Australia	Modified Delphi (Level VI)	NPs with > 12 months experience (*N* = 966) reached consensus on proposed meta specialties via a 3-round Delphi model within NP practice of emergency and acute care, primary healthcare, child and Family healthcare, and mental healthcare (response rate was *n* = 212 completed for round 1, *n* = 205 completed for round two and *n* = 197 completed for round three). Two meta specialties did not achieve consensus.	**X**	**X**	
Faraz (2017), USA	Cross-Sectional Study (Level VI)	A study of new primary care NPs (*n* = 177) found the variables most predictive of turnover intention were professional autonomy (*p =* 0.001) and role ambiguity (*p =* 0.03), which accounted for 48% of the variance in turnover intention. Quality of professional and interpersonal relationships was not a significant predictor.			**X**
Fulton, Clark (2017), USA	Observational Study (Level VI)	Primary care NP students’(*n* = 97) on clinical placement time spent 34% of their time with patients or preceptors, therefore, a competency-based skills demonstration before graduation may be a superior strategy for ensuring competency.		**X**	
Sabatino, Pruchnicki (2017), USA	Pre-test Post-test Study (Level IV)	Evaluation of a 14-week pharmacist-led online prescribing and legal considerations module found significant improvement in Family NPs (*N* = 26) error identification (+ 17%, *p* < 0.001); however, the mean performance was less than the required 70% pass rate. Whilst the intervention improved recognition and avoidance of errors, it did not improve competency.		**X**	
Aruda, Griffin (2016), USA	Observational Survey (Level VII)	Paediatric Primary Care NPs (*N* = not reported) most performed skills should be core competencies, and taught in the speciality curricula.		**X**	
Currie, Chiarella (2016), Australia	Cross-Sectional Survey (Level VI)	The 2010 expansion of MBS and PBS subsidies to privately practising NPs has increased access to care. A quarter (26%) of privately practising Australian NPs (*N* = 73) work in general practice, with most in private practice (74%) and working with other health professionals (70%) and two-thirds (67%) working outside of traditional office hours, and/or on weekends.	**X**		
Poghosyan and Liu (2016), USA	Cross-Sectional survey (Level VI)	A USA primary care NP (*N* = 314) survey found that increased NP autonomy and favourable relationships with organisational leadership significantly improve teamwork (*p* < 0.0001 and *p* < 0.001, respectively).			**X**
Xue and Intrator (2016), USA	Expert Opinion (Level VII)	Providing USA NPs full authority within their scope of practice would encourage more NPs to work in primary care, particularly in rural areas. NPs’ core competencies should include education about the risk factor profiles of vulnerable populations, which differ from the general population. Policy reforms could improve healthcare access and outcomes.			**X**
Xue, Ye (2016), USA	Systematic Review (Level V)	States with greater scope of practice authority tend to have increased workforce capacity and coverage of healthcare, particularly among vulnerable clinical populations in rural settings, compared to states with restricted/reduced scopes of practice.			**X**
Kooienga and Carryer (2015), USA & New Zealand	Expert Opinion (Level VII)	A global scope of practice would enhance NPs’ ability to transfer their credentials to other jurisdictions and conduct translational research, given the USA, Canada, New Zealand, and Australian regulatory experiences.			**X**
Hing and Hsiao (2015), USA	Secondary analysis of health record data (Level IV)	An aggregated group analysis of the workforce trends of primary care physicians (*N* = 1951) reported that NPs were likelier to work in primary care if practices were larger and more rural. There was no association between state regulation and increased use of NPs in primary care.			**X**
Budd, Wolf (2015), USA	Observational Survey (Level VI)	Most (90%) NPs graduates (*N* = 332) had completed the Family NP program. Half (48%) anticipated working in primary care, and students tended to stay in the same type of location following graduation as where they were currently working. The authors suggest harnessing the impact of mentoring and providing career planning to encourage students to consider primary care upon graduation.		**X**	
Spetz, Fraher (2015), USA	Secondary analysis of health provider data (Level IV)	Of practising NPs in North Carolina (*n* = 3972) and California (*n* = 839), most (80–90%) completed a primary care-focused NP program; only 58% of NPs in North Carolina and 68% of NPs nationally work in primary care roles.	**X**	**X**	

### Characteristics of the peer-reviewed literature

Three-quarters (73%) of the articles were from the US, where NPs are the largest providers of non-physician primary care but whose scope of practice differs according to state laws [10]. Articles from all of the included countries, except Singapore, were identified and included. Of the 79 included articles, nearly half (47%) related to NP regulation, 42% related to education of NPs and 22% to practice pathways (note that some articles applied to multiple foci) (Refer to Table 2).

The highest level of evidence (Level IV) was generated from 10 secondary analyses of service use and expenditure/billing data studies with a health economic focus [10, 11, 27–34], and one pre-test, post-test study [35]. 

### Impact of primary care nurse practitioners

Internationally, between 2002 and 2015, the growth in the NP workforce was 3–9% greater than the physician workforce [36]. This global growth reflects the increasing demand for NPs, especially in areas of unmet primary care needs [37–42]. The existing health economic evidence suggests that primary care NPs increase the communities’ access [43] to high-quality, safe and cost-effective healthcare [10, 11, 27, 44] and their patients have comparable outcomes to physician-led primary care [45, 46]. Despite the potential efficiencies of NP roles, clinical and policy stakeholders suggest that they are still often underutilised in primary care [46–48]. 

### Impact of entry pathways and program requirements on the context of NP practice

Several countries provide broad clinical NP study streams, including the US, where NP candidates looking to work in primary care complete their studies in one of the following specialty streams: Family Practice (Generalist in Primary Care); Adult-Gerontology (Primary), Paediatrics (Primary); or Women’s Health [34]. Canadian candidates study one of three streams: paediatric, adult and family streams; and in the Netherlands, candidates focus on physical or mental health.

In the US, defined NP clinical streams determine graduates’ practice context, with rural NPs more likely to have a Family NP certification [49, 50], while urban NPs are more likely to have adult or gerontology certifications [49]. A review of US medical billing and health record data found rural NPs practice more autonomously than their urban counterparts despite no significant differences in the complexity of care [29]. Whilst 70% of all US NPs have a Family Practice certification, less than one-third consider primary care their main focus, with an increasing number of NPs employed in sub-speciality ambulatory practices or inpatient units [51], effectively reducing the availability of NPs to work in primary care.

Removing pre-entry clinical experience requirements in the US has supported a rise in the number of courses accepting candidates directly from undergraduate nursing programs. Some US courses offer direct-entry that allow non-nurses to concurrently obtain their Registered Nurse and NP licensure [52], leading to an increase in the clinical practice hours required by the Doctor of Nursing Practice compared to Master’s candidates from an average of 693 to 981 h (Refer Table 1). This change has raised concerns about Registered Nurses with little or no clinical experience entering an NP course and their ability to gain capabilities to practice safely and effectively, particularly in states with full practice authority [53]. However, a recent qualitative study of primary care NPs found these less experienced NP candidates were equally competent by the end of their NP course as Registered Nurses with more clinical experience prior to entry [52]. 

In Australia, Ireland and the Netherlands there are few primary care nursing courses [37, 54, 55]. While Irish Universities are willing to expand their offerings in primary care, there is no clear path to actualisation [54]. Most NPs in Australia practice in acute care settings, which has raised concerns in response to increasing societal primary care needs [48, 56]. A modified Delphi study in Australia reported consensus among experienced NPs that primary care should be classified as a ‘meta-speciality’ and be used to guide the development of NP learning and clinical outcomes [39]. Another mixed methods Australian study reported NPs identified the areas of greatest need over the next five years, including aspects of primary care such as chronic disease, generalist and rural/remote care [40]. Only one NP in this study was identified as a primary care practitioner, illustrating the urgent need to prepare more Australian NPs to meet the countries growing primary care workforce demands.

New Zealand has introduced a policy to expand primary care services; however, NPs report numerous practice barriers, including high costs of the NP pathway, reduced funding for primary care nurses and difficulty securing placements, among others [57, 58]. Canadian studies similarly reported that improving access to funding may enhance the integration of NPs into the primary care setting [59]. They identified a lack of defined pathways to primary care roles, particularly in rural locations [60]. Difficulty obtaining placements and irregular clinic funding of primary care NP candidates were similarly flagged as barriers to primary care practice in rural US communities [61]. 

### Education of primary care nurse practitioners

#### Supervised clinical placement hours

A 2020 global comparative analysis of university programs identified an inverse relationship between the number of clinical hours required for admission and the number of clinical hours embedded in NP programs [62]. The exception is the Netherlands, which has stringent admission criteria and the most clinical placement hours (2000 h) due to government funding of the full-time employment for NP candidates while they study [62]. The web search confirmed this with Canada and the US having lower clinical experience requirements for entry to NP programs, but mandating more clinical placement hours. In contrast, Australia requires an average of 4.22 years of clinical experience to enter the program but mandates fewer placement hours (refer to Table 1).

#### Primary care skills

Many NPs working in the US and Australian primary care sector perceive their educational program was insufficient in preparing them with the clinical skills required for independent practice [37, 53, 63, 64]. In the US, there is a potential mismatch between the skills taught in the primary care curriculum and those used in NP clinical practice. For example, assessment, diagnostic investigation and interpretation are vital elements of the primary care NP role; [65, 66] however, skills such as mental health assessments [66], ordering diagnostic tests [65, 66], basic primary care procedures [64–66], ECG and X-ray interpretation [64, 65, 67], and chronic pain management [66] are inconsistently taught in primary care programs, or missing altogether. As a result, numerous articles focused on the importance of competency-based education in primary care, calling for practice standards and curricula to align with the clinical activities that typify nurse practitioners’ workloads to ensure safe and effective care [65–68]. There are emerging programs addressing these gaps, such as one reported rural primary care preceptorship for Family Nurse Practitioner students, embedding practical skill workshops into the program and providing clinical placements in rural settings, which led to 56% of participants accepting jobs in rural primary care [69]. 

The topic of competency-based versus capability-based education is a subject of international discussion. A US study found students spent only 34% of their time on placement with patients or preceptors, arguing that a competency-based demonstration of skills before graduation may be a superior strategy for measuring competency [70]. Australia has a capability-based approach, where the NP’s individual speciality shapes their clinical practice [37, 71, 72]. More recently, it has been suggested that implementing standardised education streams aligned with national health priorities (including primary health) and replacing the significant advanced practice experience hours with competency-based assessments [37]. Another recent Australian study sought industry consensus on key skills and competencies for various NP meta-specialities, including primary care, to help guide local and international NP education [38, 39]. 

Multidisciplinary and virtual education are changing how education is delivered to nurse practitioner students. Two studies examined the benefits of multidisciplinary education for primary care NPs, finding an improvement in self-efficacy was statistically significant for NPs who completed an interprofessional program with dental students compared with non-participants (*p* = 0.02) [73]. A 14-week multidisciplinary pharmacy led program improved Family NPs’ recognition and avoidance of medication errors, although overall competency was not statistically improved [35]. Virtual programs for rural primary care NP candidates [74, 75] reported participant satisfaction with virtual education, simulation and evaluation of core clinical skills in primary care, however these two studies reported incomplete methodology. More rigorous research is needed in evaluating education modalities in primary care.

#### Primary care transitional programs

Qualitative studies suggest focusing on the transition from education to practice is important and is linked to workforce retention [50, 76]. One study of novice NPs in primary care (*n* = 177) reported mentorship, professional development and role support as facilitators of this transition [50]. Further, positive clinical experience and perceptions of mentorship and preceptors were identified as some of the top predictors of NP students choosing to work in primary care [77]. As a result of growing evidence, the US National Academy of Medicine has recommended establishing accredited and standardised postgraduate training for primary care providers, including NPs [78]. Yet fewer than 10% of US primary care NPs have completed these programs [63, 79] despite participants reporting they were effective in clinical practice preparation. Residencies and fellowships are said to address the transitional challenges many US NPs experience [53, 63, 80–82] by improving their confidence [79, 81, 83, 84], clinical competencies [81, 85–87], interprofessional collaboration and communication [79, 80, 84, 86], patient outcomes [82, 83] and reducing workforce attrition [78, 79, 82, 83]. 

The data on these US NP transitional programs are relatively new, and more research is needed to determine the quality and impact of primary care residencies and fellowships [78]. While US primary care residencies are more often accredited than other specialities, there are calls to formally accredit and standardise these programs nationwide [83]. Accreditation assures professional nursing organisations’ involvement in curriculum development and learning outcomes [78]. While this review yielded results of transitional programs only from the US, two articles from Ireland [54] and Australia [81] suggested that primary care residency programs similar to those offered in the US could be beneficial for NP role-preparation.

### Regulation of primary care nurse practitioners

#### Full practice authority in primary care

Much of the literature on regulation is US-centred, with ongoing conversations relating to full practice authority in primary care. In the US, the scope of NPs practice reflects state regulations, which have either full, reduced or restricted practice authority, determining how independently an NP can practice [32]. Much of the literature suggests that full-practice authority is required for primary care NPs if they are to: improve patient access to primary care [28, 30, 33, 46], particularly in low socioeconomic and rural areas [10, 41, 42], reduce hospitalisations [27, 44], and reduce healthcare and training costs [1, 11, 33, 44, 88]. Strong organisational support for independent practice increases NP’s capacity to provide effective primary care, and improves teamwork among NPs and physicians [89–91]. States with full authority reported a higher proportion of adults reporting an NP as their main primary care provider [31]. For workforce planning purposes, autonomous practice was associated with a reduction in turnover intention reported by primary care NPs [92]. Only one study reported no association between state regulation and increased use of NPs in primary care [93]. 

Variations in US state and organisational regulations (e.g., supervision requirements), especially in states with restricted and reduced practice authority, pose significant barriers to entry to primary practice, one study reported NPs were 13% more likely to practice in primary care in states with a full scope of practice [94]. These variations also limit NPs’ capacity to practice to the full extent of their qualifications [47, 95–98] and across jurisdictions [99]. These restrictions may also increase healthcare costs due to increased service and provider fees [94]. While the number of registered NPs is rising, states with reduced or restricted authority have the largest care gaps in identified primary care shortage areas and rural communities compared with states with full practice authority [32]. 

#### The transition from master to doctor of nursing practice programs in the US – implications for primary care

The move to the Doctor of Nursing Practice in the US by 2025 [100] has implications for the primary care workforce. Early evidence suggest that Doctor of Nursing Practice NPs are likely to move directly into leadership, policy or management instead of direct care roles, impacting NP workforce availability and planning [100–103]. One study identified that only 11% of Doctor of Nursing Practice graduates practice in primary care [101], while another reported that 85% of Doctor of Nursing Practice programs in 2018 were non-clinical, focusing on leadership and administration [103]. These findings have led to concerns within the sector that this change impacts NP roles, and if this trend continues, it may impact the US’s ability to grow its primary care workforce [103]. 

#### Non-US perspectives on NP regulation in primary care

While there were few non-US studies, a recurring theme was the ambiguous role of primary care NPs within the health system due to ineffective or insufficient policy and governance [54, 55, 59, 72]. For example in the Netherlands, while the NP hospital role is well established, integrating NPs into primary care is relatively new, with suggestions that a lack of international guidance has prevented standardising the NP role and created role confusion [55]. 

Similarly in Ireland, the *Slάintecare* policy was developed to increase NP services in areas of need, including primary care. When the definitions of primary care and the NP role were identified as being unclear, a national project sought international consultation to help define the role, leading to the development of robust regulation and postgraduate continuing education for NPs [54]. 

In Alberta, Canada, the NP Support Program policy was designed to integrate NPs into the primary care system but is said to have failed due to a lack of clear role delineation within primary care [59]. Stakeholders also reported that this policy had inadvertently limited NPs’ job opportunities, embedded financial disincentives and promoted physician gatekeeping, impeding NPs’ ability to practice independently in Alberta [59]. 

In Australia, a recent study reported limited advocacy from employers and policy advisors for expanding the NP scope of practice or increasing payment for NP services, suggesting NPs must lobby themselves for regulation changes [72]. Australian and New Zealand primary care NPs face constraints such as restricted items they can claim for government reimbursement through universal healthcare funding, or inability to sign off on vital certifications (e.g. work cover and time off work, death certification), effectively limiting autonomous practice [56, 104]. Despite evidence that NP-led primary care has cost benefits to the health system, reduces hospitalisations and improves early health interventions; policy and legislation in Australia restrict primary care NPs from exercising their full scope of practice [105]. Overall, the international literature and stakeholder feedback calls for better alignment of funding, policy and governance structures to ensure improved integration of NP practice into primary care [54, 55, 58, 59]. 

## Discussion

Despite most of the included articles coming from the US, a country without universal healthcare, several significant findings have emerged from this scoping review that have implications for shaping the global primary care NP workforce. Two distinct NP entry, regulation, and practice pathways have emerged from this scoping review: (1) pre-defined clinical streams versus (2) bespoke clinical expertise.

Globally, there are marked differences in the NP entry requirements, ranging from a postgraduate diploma to new graduates with no clinical experience entering a 3-4-year NP program. In Australia, Ireland, New Zealand, the Netherlands and Singapore, NP candidates must have practised and demonstrated competencies in their chosen sub-speciality, which may include primary care [106]. While countries like the US and Canada have a defined primary care stream, countries such as Australia, New Zealand, Ireland, and the Netherlands lack this definition, with no clearly defined primary care pathways. These countries rely on individual nurses with demonstrated primary care expertise electing to progress into an NP program. While Australia is the only country requiring a postgraduate certificate for NP program admission, Australian and Singaporean NP programs [107–109] require substantial full-time equivalent clinical experience in the candidates chosen speciality, which means that candidates tend to enter at an older age, compared to the US.

These differing NP entry pathways reflect each country’s NP workforce profile, including primary care. US and Canadian NPs are younger nurses undertaking more extended clinical supervision in varying practice environments within broad population-based groups in a defined clinical stream, including primary care [62]. Whereas Australian nurses entering an NP program have significant clinical experience in their chosen speciality or sub-speciality, with an individualised scope of practice, undertake fewer supervised clinical placement hours, and, as a result, tend to be older [62]. Few currently have primary healthcare experience because there are fewer primary healthcare nursing programs, and primary healthcare roles are harder to secure, making it difficult for registered nurses to demonstrate their specialist expertise within Australia’s current fee-for-service primary healthcare system [104]. This reality has led to calls for Australia to move from its individualistic specialist entry requirement approach to standardised NP speciality streams aligned with national health priorities, such as primary care [37, 38]. Similar to the focused Canadian specialities, where candidates enter one of three streams: paediatric, adult and family streams, or the Netherlands, where candidates choose to focus on physical or mental health. Considering different entry pathways will be challenging for international health systems, but critical if the NP workforce is to play a more significant role in caring for the growing needs of people living with chronic and other unmet primary healthcare needs.

The findings suggest that building the primary care NP workforce requires targeted whole-of-sector strategies. At a systems level, specialist entry requirements, clinical practice hours and access to reimbursement items or a financial model are critical to supporting NPs to practice in primary care [104]. At the organisational level, the practice environment can serve as a facilitating factor, as evidenced by US rural NPs, who are more likely than their urban counterparts to manage their patient care as primary care providers independently [29, 49, 110]. At a personal level, professional development opportunities, institutional commitment to ongoing education, work-life balance, mentorship, autonomy, ability to use clinical assessment and decision-making skills [57, 77, 90, 98] are additional factors that help attract and keep NPs in primary care practice.

While countries like Australia have adopted a capability framework [111], other countries, including the US, have adopted a competency framework to prepare NPs for practice [112]. There have been suggestions that it might be time to revisit the sector’s need for a suite of generic NP competencies, ensuring that all NPs demonstrate standardised foundational competencies across their speciality [37–40]. However, the literature suggests the skills necessary for autonomous practice in primary care are not consistently incorporated into NP curriculum, and NP candidates often report feeling inadequately prepared for autonomous practice after registration. Any future competency-based frameworks need to address the skills required in primary care practice.

Despite few being accredited, US transitional primary care residency and fellowship participants report favourable outcomes [83, 103]. Regulation and accreditation of US transitional primary care NP programs would provide a blueprint for other countries to adopt and may help reduce the attrition of NPs from primary care [54, 78, 81, 83]. 

While it is premature to evaluate the impact of the introduction of the US’s Doctor of Nursing Practice by 2025, on NPs choosing primary care, early data does suggest a larger proportion of Doctor of Nursing Practice graduates take up administrative or leadership positions compared to clinical roles [100, 101, 103]. This is relevant considering US workforce projections indicate an ongoing decline in the primary care NP and physician-to-population ratio [9]. If this trend continues, it may adversely impact the global need for more clinically focused primary care NPs as demand increases.

Numerous barriers exist to practising as a primary care NP. Several US studies identified common barriers to choosing primary care as a speciality, including obtaining clinical placements, a poor understanding of the primary care NP role, and legally mandated NP supervision by physicians [61, 94, 98]. Further, some US states remain constrained by restrictive practices which prevent them from performing to their full scope of practice [1, 9, 41, 42, 47, 94, 95, 97–99]. Australia has traditionally had a system of collaborative arrangements in place in primary care practice, where NPs were required to be effectively supervised by a physician, not unlike a similar practice that exists in some US states [97]. This widely criticised practice affects NPs’ ability to practice independently without an arrangement and to claim Medicare rebates and Pharmaceutical Benefits Scheme items [97, 105]. The Australian Government’s recent commitment to remove the mandated requirement for a collaborative arrangement will support Australian primary care NPs to work more autonomously. Independent practice in primary care is an important discussion, as reflected in the Australian NP standards for practice, which specify that NPs can effectively manage care episodes as the primary provider [111]. However, the rise in complex chronic diseases (including heart disease, diabetes, COPD, progressive neurological conditions and increasingly cancer survivorship) [17] means interdisciplinary health teams are necessary to address the complex needs of many primary care patients [113]. Nurse Practitioners are ideally positioned to work as part of these interdisciplinary teams to optimise care for people with chronic and complex conditions [111]. 

Despite these enormous opportunities, Irish and Australian nurses have limited opportunities within the current educational pathways to build their primary care capabilities [37, 54]. Cultural perceptions of NP-led care as disparate to physician-led care in New Zealand and the Netherlands were identified as barriers, compounded by difficulties completing prescribing practicums, fewer scholarships, personal costs associated with completing a Master’s, and difficulty securing employment as a primary care NP [55, 57]. The policies implemented in Canada and New Zealand with the aim of enhancing the integration of NPs into primary care have inadvertently yielded unfavourable outcomes, such as disparities in funding, policies fostering competition between NPs and Physicians, insufficient opportunities for job creation in primary care and restricted reimbursement items, which, collectively, have impeded the potential growth of NPs in primary care settings [58, 59]. Expanding the workforce in primary care will continue to be a challenge while these barriers exist. Including NP and relevant stakeholder voices in policy consultation is recommended to ensure the goals are operationally viable and beneficial to NPs.

## Limitations

There are several limitations and strengths associated with this rapid review. Only including studies published since 2015 may have excluded earlier seminal work. However, the most significant limitation is that most of the included studies generated low-level evidence, making it challenging to draw any definite conclusions, especially as there was no quality appraisal of the evidence as part of the rapid review methodology. Most of the included studies also reported on the US experience, which differs considerably from other countries’ healthcare and regulatory systems. As it was not feasible to review all current US NP program requirements, restricting the appraisal to 30 US NP programs may not accurately reflect the full scope of the available NP programs. This was balanced by including diverse programs from different states. Despite these limitations, this review has considerable strengths. It canvased material from multiple sources, including 86 NP programs, and evidence from the current English peer-reviewed literature to provide a detailed global snapshot of NP regulation, education, and practices and how it enables or restricts the development of the NP primary care role.

## Conclusions

Globally, NP roles continue to grow in both numbers and stature. The variations in the entry pathways, accreditation, education, endorsement or licensure, and professional pathways available to NPs across the US, Canada, Australia, New Zealand, Ireland, Singapore and the Netherlands reflect the local needs, changing circumstances and different regulations. Differing entry and practice requirements shape the composition and function of each country’s NP workforce, including age, clinical focus and expertise. These requirements ultimately influence where NPs practice and the populations they serve, including in primary care. It may be timely for countries who wish to grow their primary healthcare workforce to (1) revisit the merits of introducing a non-specialist NP entry pathway that attracts high-quality nurses interested in addressing national health priorities and providing care to underserved communities; (2) consider the limitations of restricted practice and economic implications of removing restrictions and (3) ensuring primary care NP curricula is informed by real-world skills and practice.

### Electronic supplementary material

Below is the link to the electronic supplementary material.

Supplementary Material 1

Supplementary Material 2

## Data Availability

No datasets were generated or analysed during the current study.

## Abbreviations

DNP: Doctor of Nursing Practice
NP: Nurse Practitioner
US: United States
