# Supplementary material for: Practice pathways, education, and regulation influencing nurse practitioners’ decision to provide primary care: a rapid scoping review
Source: BMC Prim Care. 2024 May 23;25:182. doi: 10.1186/s12875-024-02350-3 (PMC11112961; doi:10.1186/s12875-024-02350-3)
Supplement: Supplementary file 1 — Supplementary Material 1 [file 12875_2024_2350_MOESM1_ESM.docx]

### Additional File 1

Search strings used to identify peer-reviewed publications for inclusion in the rapid literature review:

| **Search engine** |  | **Search string** |  | **Citations** |
| --- | --- | --- | --- | --- |
| Medline |  | AB (NP OR np) AND ((education OR accreditation OR endorsement OR regulation) OR (‘entry requirements’  OR pathway OR 'admission requirement') OR ('primary care' OR generalist)) |  | 4,879 |
| CINAHL |  | AB (NP OR np) AND ((education OR accreditation OR endorsement OR regulation) OR ('entry requirements'  OR pathway OR 'admission requirement') OR ('primary care' OR generalist)) | | 2,493 |
