# Supplementary material for: Practice pathways, education, and regulation influencing nurse practitioners’ decision to provide primary care: a rapid scoping review
Source: BMC Prim Care. 2024 May 23;25:182. doi: 10.1186/s12875-024-02350-3 (PMC11112961; doi:10.1186/s12875-024-02350-3)
Supplement: Supplementary file 2 — Supplementary Material 2 [file 12875_2024_2350_MOESM2_ESM.docx]

Additional File 2

Table 1: Nurse Practitioner Programs from Australia, Canada and Ireland identified in the web-search

| **Australia**  **(n=9)** |  | **Canada**  **(n=21)** | |  | **Ireland**  **(n=8)** |
| --- | --- | --- | --- | --- | --- |
| Charles Darwin University (2022) |  | Lakehead University (2022) | University of Northern British Columbia (2022) |  | Munster Technological University and School of Health and Social Science (nd) |
| Edith Cowan University (2022) |  | Lakehead University (2022) | University of Ottawa (nd) |  | NUI Galway School of Nursing and Midwifery (2022) |
| LaTrobe University (2022) |  | Laurentian University (2021) | University of Victoria (2021) |  | RCSI School of Nursing and Midwifery (nd) |
| Queensland University of Technology (2022) |  | McMaster University (2017) | University of Windsor (nd) |  | Waterford Institute of Technology School of Health Sciences (2022) |
| The University of Newcastle (2022) |  | McMaster University (2017) | University of Windsor (nd) |  | Trinity College Dublin School of Nursing and Midwifery (2021) |
| The University of Sydney (2022) |  | Queens University (2017) | Western University (2022) |  | UCC School of Nursing and Midwifery (2021) |
| University of Melbourne (2022) |  | Queens University (2017) | Western University (2022) |  | UCD School of Nursing (2016) |
| University of Queensland (2022) |  | Ryerson University (nd) | York University (nd) |  | University of Limerick Department of Nursing and Midwifery (nd) |
| University of South Australia (2022) |  | Univeristy of Toronto (2022) |  |  |  |
|  |  | University of British Columbia (2022) |  |  |  |

Table 2: Nurse Practitioner Programs from the Netherlands, New Zealand and Singapore identified in the web-search

| **Netherlands**  **(n=10)** |  | **New Zealand**  **(n=7)** |  | **Singapore**  **(n=1)** |
| --- | --- | --- | --- | --- |
| Fontys (nd) |  | Ara Institute of Canterbury (2022) |  | National University of Singapore (nd) |
| GGZ-VZ (nd) |  | Auckland University of Technology (2022) |  |  |
| Han University of Applied Sciences (nd) |  | Eastern Institute of Technology (2022) |  |  |
| Hanze University of Applied Sciences (2020) |  | Massey University (2022) |  |  |
| Hogeschool Leiden (nd) |  | University of Otago (nd) |  |  |
| Inholland University of Applied Sciences (2022) |  | Victoria University of Wellington (2022) |  |  |
| Rotterdam University of Applied Sciences (2022) |  | Waikato Institute of Technology (nd) |  |  |
| Saxion (2022) |  |  |  |  |
| University of Applied Sciences Utrecht (2022) |  |  |  |  |
| Zuyd (nd) |  |  |  |  |

Table 3: Nurse Practitioner Programs from the United States of America - the web-search

| **US**  **(n=30)** | | |
| --- | --- | --- |
| Carolina Nursing (2022) | Penn Nursing (2022) | UCSF School of Nursing, (2021) |
| Columbia School of Nursing (2022) | Rush University College of Nursing (2022) | University of Mary (2022) |
| Delta State University Robert E. Smith School fo Nursing (2022) | Rutgers School of Nursing (2022) | University of Michigan School of Nursing ( |
| Duke University School of Nursing (2022) | South Dakota State University College of Nursing (2022) | University of Vermont College of Nursing and Health Sciences (2022) |
| Frances Payne Bolton School of Nursing (2022) | The Ohio State University College of Nursing (2022) | University of Washington School of Nursing (2022) |
| Henderson State University Ellis College of Arts and Sciences (2022) | The University of Kansas School of Nursing (2022) | UVA School of Nursing (2022) |
| Husson University School of Nursing (2022) | The University of Texas at Austin School of Nursing (2022) | Vanderbilt School of Nursing (nd) |
| Johns Hopkins University School of Nursing (2022) | UAB School of Nursing (2022) | Wesleyan College of Nursing (nd) |
| Montana State University Mark and Robyn Jones College of Nursing (nd) | UCLA School of Nursing (2022) | Western Kentucky University School of Nursing and Allied Health (2021) |
| Nell Hodgson Woodruff School of Nursing (2021) | UIC College of Nursing (2022) | Yale University School of Nursing (2022) |

1. Charles Darwin University. Master of Nursing (Nurse Practitioner) 2022 [Available from: <https://www.cdu.edu.au/study/course/master-nursing-nurse-practitioner-snnp01>.

2. Lakehead University. Nursing- Nurse Practitioner Specialization 2022 [Available from: <https://www.lakeheadu.ca/programs/departments/health-sciences/graduate-programs/master-of-public-health-programs/program-options/mph-with-specialization-in-nursing-with-nurse-practitioner-electives>.

3. University of Northern British Columbia. Family Nurse Practitioner 2022 [Available from: <https://www2.unbc.ca/nursing/family-nurse-practitioner-mscn-fnp>.

4. Munster Technological University, School of Health and Social Science. MTU Course Filter Options: Munster Technological University; [Available from: <https://www.ittralee.ie/en/InformationAbout/Courses//?listStudy=NURS&listStudylevel=PG&coursetypeSubject=all>.

5. Edith Cowan University. Master of Nursing (Nurse Practitioner) Handbook: Course Information 2022 [Available from: <https://www.ecu.edu.au/handbook/course?id=L88&year=2022>.

6. Lakehead University. Nursing- Nurse Practitioner Specialization 2022 [Available from: <https://www.lakeheadu.ca/programs/graduate/programs/masters-specialization/nursing-practitioner/node/62514#requirements>.

7. University of Ottawa. Graduate Diploma Primary Health Care for Nurse Practitioners nd [Available from: <https://catalogue.uottawa.ca/en/graduate/diploma-primary-health-care-nurse-practitioners/>.

8. NUI Galway School of Nursing and Midwifery. Masters/Postgraduate Diploma: National University of Ireland, Galway; 2022 [Available from: <https://www.nuigalway.ie/medicine-nursing-and-health-sciences/nursing/postgraduate/masterspostgraduatediploma/>.

9. LaTrobe University. Master of Nursing (Nurse Practitioner) Online- Handbook 2022 [Available from: <https://handbook.latrobe.edu.au/courses/2022/HMNNPO>.

10. Laurentian University. Nurse Practitioner 2021 [Available from: <https://laurentian.ca/program/nursing-phcnp/details>.

11. University of Victoria. Nurse Pracitioner Option 2021 [Available from: <https://www.uvic.ca/hsd/nursing/graduate/practitioner/index.php>.

12. RCSI School of Nursing and Midwifery. Advanced Practice Nursing: Royal College of Surgeons in Ireland: University of Medicine and Health Sciences; [Available from: <https://www.rcsi.com/dublin/postgraduate/taught-courses/advanced-practice-nursing-msc/overview>.

13. Queensland University of Technology. Master of Nurse Practitione 2022 [Available from: <https://www.qut.edu.au/courses/master-of-nurse-practitioner>.

14. McMaster University. MSc Course Based PHCNP 2017 [Available from: <https://nursing.mcmaster.ca/programs/graduate/msc-course-based-phcnp>.

15. University of Windsor. Master of Nursing. nd [Available from: <https://www.uwindsor.ca/nursing/317/master-nursing-degree>.

16. Waterford Institute of Technology School of Health Sciences. Master of Science Nursing: Waterford Institute of Technology; 2022 [Available from: <https://www.wit.ie/courses/msc_in_nursing_part_time#overview>.

17. The University of Newcastle. Master of Nurse Practitioner Program Handbook 2022 [Available from: <https://www.newcastle.edu.au/degrees/master-nurse-practitioner/handbook>.

18. McMaster University. PHCNP Graduate Diploma 2017 [Available from: <https://nursing.mcmaster.ca/programs/graduate/phcnp-graduate-diploma>.

19. University of Windsor. Graduate Diploma - Primary Health Care Nurse Practitioner nd [Available from: <https://www.uwindsor.ca/nursing/318/graduate-diploma-primary-health-care-nurse-practitioner>.

20. Trinity College Dublin School of Nursing and Midwifery. Nursing-Advanced Practice (ANP): Trinity College Dublin; 2021 [Available from: <https://nursing-midwifery.tcd.ie/postgraduate/taught-masters/nursing-anp>.

21. The University of Sydney. Master of Nursing (Nurse Practitioner) 2022 [Available from: <https://www.sydney.edu.au/courses/courses/pc/master-of-nursing-nurse-practitioner.html>.

22. Queens University. Master of Nursing (Primary Healthcare Nurse Practitioner) 2017 [Available from: <https://nursing.queensu.ca/graduate/master-nursing-mnphcnp>.

23. Western University. Primary Health Care Nurse Practitioner Diploma (PHCNP) 2022 [Available from: <https://www.uwo.ca/fhs/nursing/graduate/course_based/phcnp/index.html>.

24. UCC School of Nursing and Midwifery. UCC Postgraduate Courses Nursing - Advanced Practice. : University College Cork, Ireland; 2021 [Available from: <https://www.ucc.ie/en/ckx24/>.

25. University of Melbourne. Master of Advanced Nursing Practice (Nurse Prctitioner) 2022 [Available from: <https://study.unimelb.edu.au/find/courses/graduate/master-of-advanced-nursing-practice-nurse-practitioner/>.

26. Queens University. Primary Healthcare Nurse Practitioner Diploma 2017 [Available from: <https://nursing.queensu.ca/graduate/phcnp-diploma>.

27. Western University. Master of Nursing – PHCNP (MN-PHCNP) 2022 [Available from: <https://www.uwo.ca/fhs/nursing/graduate/course_based/mnphcnp/index.html>.

28. UCD School of Nursing MaHS. Advanced Practice Nursing: University College Dublin; 2016 [Available from: <https://www.nmhs.ucd.ie/study-with-us/graduate-programmes/advanced-practice-nursing>.

29. University of Queensland. Master of Nurse Practitioner 2022 [Available from: <https://future-students.uq.edu.au/study/programs/master-nurse-practitioner-5677>.

30. Ryerson University. Combined Master of Nursing/Primary Health Care Nurse Practitioner Certificate (MN/PHCNP) nd [Available from: <https://www.ryerson.ca/nursing/graduate/combined-mn-phcnp-certificate/>.

31. York University. Primary Health Care Nurse Practitioners (PHCNP) nd [Available from: <https://www.yorku.ca/gradstudies/nursing/programs/phcnp/>.

32. University of Limerick Department of Nursing and Midwifery. Postgraduate Programmes: University of Limerick; [Available from: <https://www.ul.ie/nursing-midwifery/programmes/postgraduate-programmes>.

33. University of South Australia. Master of Nursing (Nurse Practitioner) 2022 [Available from: <https://study.unisa.edu.au/degrees/master-of-nursing-nurse-practitioner>.

34. Univeristy of Toronto. Bachelor of Science in Nursing (BScN) Nurse Practitioner 2022 [Available from: <https://bloomberg.nursing.utoronto.ca/programs/master/nurse-practitioner/#content4>.

35. University of British Columbia. Master of Nursing- Nurse Practitioner 2022 [Available from: <https://nursing.ubc.ca/graduate-learning/master-nursing-nurse-practitioner>.

36. Fontys. Advanced Nursing Practice (master) nd [Available from: <https://fontys.nl/Professionals-werkgevers/Opleidingen-en-cursussen/Advanced-Nursing-Practice-master.htm>.

37. Ara Institute of Canterbury. Master of Nursing Pathway for Nurse Practitioners; Frequently Asked Questions 2022 [Available from: <https://www.ara.ac.nz/siteassets/documents---home/study/study-interest-areas/nursing-midwifery-and-medical-imaging/nursing/04349-mn-np-faq-sheet-a4.pdf>.

38. National University of Singapore. Master of Nursing nd [Available from: <https://medicine.nus.edu.sg/nursing/education-2/postgraduate/master-of-nursing/>

39. GGZ-VZ. The Training- Practical Information nd [Available from: <https://ggzvs.nl/de-opleiding/wat-doen-we/praktische-informatie/>.

40. Auckland University of Technology. Advanced Nursing Practice- Master of Health Science 2022 [Available from: <https://www.aut.ac.nz/study/study-options/health-sciences/courses/master-of-health-science/advanced-nursing-practice>

41. Han University of Applied Sciences. Master Advanced Nursing Practice dual nd [Available from: <https://www.han.nl/opleidingen/master/advanced-nursing-practice/duaal/>.

42. Eastern Institute of Technology. Master of Nursing (Nurse Practitioner) 2022 [Available from: <https://www.eit.ac.nz/programmes/master-of-nursing/>.

43. Hanze University of Applied Sciences. Master Advanced Nursing Practice 2020 [Available from: <https://www.hanze.nl/nld/onderwijs/gezondheid/academie-voor-verpleegkunde/opleidingen/master/master-advanced-nursing-practice/studiekeuze/over-de-studie/master>.

44. Massey University. Master of Nursing 2022 [Available from: <https://www.massey.ac.nz/massey/learning/programme-course/programme.cfm?prog_id=93017#>.

45. Hogeschool Leiden. Master Advanced Nursing Practice- dual nd [Available from: <https://www.hsleiden.nl/master-advanced-nursing-practice>.

46. University of Otago. Masters of Advanced Nursing Practice nd [Available from: <https://www.otago.ac.nz/christchurch/departments/nursing/madvnp/>.

47. Inholland University of Applied Sciences. Study Programme Master of Advanced Nursing Practice 2022 [Available from: <https://www.inholland.nl/opleidingen/master-of-advanced-nursing-practice/de-opleiding/>.

48. Victoria University of Wellington. National Nurse Practitioner Training Programme (NPTP), 2022 [Available from: <https://www.wgtn.ac.nz/health/study/postgraduate/nurse-practitioner-training-programme-nptp>.

49. Rotterdam University of Applied Sciences. Advanced Nursing Practice dual 2022 [Available from: <https://www.hogeschoolrotterdam.nl/opleidingen/master/advanced-nursing-practice/duaal/>.

50. Waikato Institute of Technology. Master of Nursing (Level 9) nd [Available from: <https://www.wintec.ac.nz/study-at-wintec/courses/health-and-wellbeing/nursing/master-of-nursing>

51. Saxion. Master Advanced Nursing Practice (dual) 2022 [Available from: <https://www.saxion.nl/opleidingen/deeltijd/master/master-advanced-nursing-practice-duaal/toelating>.

52. University of Applied Sciences Utrecht. Master of Advanced Nursing Practice dual 2022 [Available from: <https://www.hu.nl/deeltijd-opleidingen/master-advanced-nursing-practice-duaal>.

53. Zuyd. Master Advanced Nursing Practice nd [Available from: <https://www.zuyd.nl/opleidingen/master-advanced-nursing-practice>.

54. Carolina Nursing. Academic Programs: The University of North Carolina at Chapel Hill; 2022 [Available from: <https://nursing.unc.edu/education/academic-programs>.

55. Penn Nursing. Master of Science in Nursing (MSN): University of Pennsylvannia; [Available from: <https://www.nursing.upenn.edu/academics/master-of-science-in-nursing-msn/>.

56. Columbia School of Nursing. Doctor of Nursing Practice: Columbia University; 2022 [Available from: <https://www.nursing.columbia.edu/academics/academic-programs/doctor-nursing-practice>.

57. Rush University College of Nursing. Nursing Programs and Admissions: Rush University; 2022 [Available from: <https://www.rushu.rush.edu/college-nursing/programs-admissions>.

58. University of Mary. BSN to DNP: Family Nurse Practitioner: University of Mary; 2022 [Available from: <https://online.umary.edu/academics/masters-doctoral-programs/bsn-dnp-family-nurse-practitioner>.

59. Delta State University Robert E. Smith School fo Nursing. Post-Master’s Certificate (Online): Delta State University; 2022 [Available from: <https://www.deltastate.edu/school-of-nursing/post-masters-certificate/#>.

60. Rutgers School of Nursing. Graduate Programs 2022 [Available from: <https://nursing.rutgers.edu/academics-admissions/graduate/>.

61. University of Michigan School of Nursing. Academics: university of Michigan - Ann Arbor; [Available from: <https://nursing.umich.edu/academics>.

62. Duke University School of Nursing. MSN Majors: Duke University; 2022 [Available from: <https://nursing.duke.edu/academic-programs/msn-master-science-nursing/msn-majors>.

63. South Dakota State University College of Nursing. Explore our Nursing Degrees: South Dakota State University; 2022 [Available from: <https://www.sdstate.edu/nursing>.

64. University of Vermont College of Nursing and Health Sciences. Doctor of Nursing Practice: University of Vermont; 2022 [Available from: <https://www.uvm.edu/cnhs/nursing/doctor_nursing_practice>.

65. Frances Payne Bolton School of Nursing. Acadmic Programs: Case Western Reserve University; 2022 [Available from: <https://case.edu/nursing/programs/>.

66. The Ohio State University College of Nursing. Academics: The Ohio State University; 2022 [Available from: <https://nursing.osu.edu/academics>.

67. University of Washington School of Nursing. Degree Programs: University fo Washington; 2022 [Available from: <https://nursing.uw.edu/programs/degree-programs-tracks/>.

68. Henderson State University Ellis College of Arts and Sciences. Graduate Nursing Programs: Henderson State University; 2022 [Available from: <https://www.hsu.edu/pages/academics/ellis-college-of-arts-and-sciences/nursing/master-of-science-in-nursing/>.

69. The University of Kansas School of Nursing. Doctor of Nursing Practice: University of Kansas; 2022 [Available from: <https://www.kumc.edu/school-of-nursing/academics/degree-programs/doctor-of-nursing-practice.html>.

70. UVA School of Nursing. Academics: University of Virginia; 2022 [Available from: <https://www.nursing.virginia.edu/academics/>.

71. Husson University School of Nursing. Graduate Nursing: Husson University; 2022 [Available from: <https://www.husson.edu/college-of-health-and-pharmacy/school-of-nursing/graduate-nursing/>.

72. The University of Texas at Austin School of Nursing. Academics: The University of Texas at Austin; 2022 [Available from: <https://nursing.utexas.edu/academics>.

73. Vanderbilt School of Nursing. Academic Programs/Specialties: Vanderbilt University; [Available from: <https://nursing.vanderbilt.edu/academic_programs/index.php>.

74. Johns Hopkins University School of Nursing. Programs and Academics: Johns Hopkins University; 2022 [Available from: <https://nursing.jhu.edu/academics/index.html>.

75. UAB School of Nursing. Academic Programs: The University of Alabama at Birmingham; 2022 [Available from: <https://www.uab.edu/nursing/home/academics>.

76. Wesleyan College of Nursing. ACADEMICS Graduate Nursing Programs: West Virginia Wesleyan College; [Available from: <https://www.wvwc.edu/graduate-nursing-programs/>.

77. Montana State University Mark and Robyn Jones College of Nursing. Degrees: Montana State University; [Available from: <https://www.montana.edu/nursing/degrees/index.html>.

78. UCLA School of Nursing. MSN - Advanced Practice Registered Nurse (APRN): University of California, Los Angeles; 2022 [Available from: <https://nursing.ucla.edu/admissions/graduate/msn-advanced-practice-registered-nurse-aprn>.

79. Western Kentucky University School of Nursing and Allied Health. College of Health and Human Sciences: Western Kentucky University; 2021 [Available from: <https://www.wku.edu/chhs/>.

80. Nell Hodgson Woodruff School of Nursing. APRN Specialties: Emory University; 2021 [Available from: <https://www.nursing.emory.edu/academics-and-admissions#Section-4>.

81. UIC College of Nursing. Doctor of Nursing Practice University of Illinois Chicago; 2022 [Available from: <https://nursing.uic.edu/programs/doctor-nursing-practice/>.

82. Yale University School of Nursing. Academics: Yale University; 2022 [Available from: <https://nursing.yale.edu/academics>.
